# Enhancing Predictive Power: Integrating a Linear Support Vector Classifier with Logistic Regression for Patient Outcome Prognosis in Virtual Reality Therapy for Treatment-Resistant Schizophrenia

**DOI:** 10.3390/jpm13121660

**Published:** 2023-11-28

**Authors:** Alexandre Hudon, Mélissa Beaudoin, Kingsada Phraxayavong, Stéphane Potvin, Alexandre Dumais

**Affiliations:** 1 Centre de Recherche de l’Institut Universitaire en Santé Mentale de Montréal, Montreal, QC H1N 3V2, Canada; alexandre.hudon.1@umontreal.ca (A.H.); melissa.beaudoin.1@umontreal.ca (M.B.); stephane.potvin@umontreal.ca (S.P.); 2 Department of Psychiatry and Addictology, Faculty of Medicine, Université de Montréal, Montreal, QC H3T 1J4, Canada; 3 Services et Recherches Psychiatriques AD, Montreal, QC H1N 3V2, Canada; kingsada@me.com; 4 Institut National de Psychiatrie Légale Philippe-Pinel, Montreal, QC H1C 1H1, Canada

**Keywords:** precision medicine, virtual reality therapy, auditory hallucinations, schizophrenia, machine learning

## Abstract

(1) Background: Approximately 30% of schizophrenia patients are known to be treatment-resistant. For these cases, more personalized approaches must be developed. Virtual reality therapeutic approaches such as avatar therapy (AT) are currently undergoing investigations to address these patients’ needs. To further tailor the therapeutic trajectory of patients presenting with this complex presentation of schizophrenia, quantitative insight about the therapeutic process is warranted. The aim of the study is to combine a classification model with a regression model with the aim of predicting the therapeutic outcomes of patients based on the interactions taking place during their first immersive session of virtual reality therapy. (2) Methods: A combination of a Linear Support Vector Classifier and logistic regression was conducted over a dataset comprising 162 verbatims of the immersive sessions of 18 patients who previously underwent AT. As a testing dataset, 17 participants, unknown to the dataset, had their first immersive session presented to the combinatory model to predict their clinical outcome. (3) Results: The model accurately predicted the clinical outcome for 15 out of the 17 participants. Classification of the therapeutic interactions achieved an accuracy of 63%. (4) Conclusion: To our knowledge, this is the first attempt to predict the outcome of psychotherapy patients based on the content of their interactions with their therapist. These results are important as they open the door to personalization of psychotherapy based on quantitative information about the interactions taking place during AT.

## 1. Introduction

### 1.1. Schizophrenia and Treatment Resistance

A recent study estimated that, in 2019, about 418 million disability-adjusted life years are caused by mental disorders, with a worldwide economic burden evaluated at USD 5 trillion [1]. Amongst mental health disorders, schizophrenia has a relatively small prevalence of less than 1% but significantly contributes to this global burden of mental disorders [2,3]. This psychotic disorder was originally defined by Eugen Bleuler in 1908 [4,5]. Schizophrenia is a functional psychotic condition marked by the presence of delusional beliefs, hallucinations, and disruptions in cognition, perception, and behavior [6,7]. Hallucinations are more often auditory in schizophrenia and are also part of an ensemble of symptoms known as positive symptoms [8,9]. This constellation of symptoms includes hallucinations, delusions, and cognitive impairments. This medical condition is not benign considering that people with schizophrenia often have a life expectancy reduction of 10 to 25 years because they have a greater suicide risk and more physical health issues than the general population [10]. Moreover, there is a higher risk of violence when the symptoms are not addressed, including hetero-aggression, victimization, and self-harm [11,12]. Indeed, violent offenses, including homicides, are also more likely in schizophrenia and other psychotic disorders [13]. However, most of the extra risk appears to be mitigated by concomitant drug usage [14,15,16]. Patients suffering from schizophrenia and benefiting from treatment usually reduce their risk of self-harm and violence [17,18]. They also experience improvements in their quality of life and life expectancy [19].

In schizophrenia, the treatment course usually includes psychopharmacological and psychotherapeutic approaches [20,21]. As dopamine is a relevant neurotransmitter involved in the positive symptoms observed in schizophrenia, the psychopharmacological component most often includes dopaminergic receptor antagonists, also known as antipsychotics [22,23]. However, about 30% of people with schizophrenia will not respond adequately to these medications and will be referred to as treatment-resistant schizophrenia (TRS) patients [24]. For those patients, most clinical guidelines support that a failure to respond to two antipsychotics warrants a trial of clozapine: a second-generation antipsychotic [25]. This approach is the current standard treatment for patients suffering from TRS. However, it is estimated that 40–70% of patients with TRS have persistent symptoms despite clozapine use [26,27]. Such symptoms include persistent auditory hallucinations, which represent the most prevalent and disabling symptoms in schizophrenia. Trials of cognitive behavioral therapy (CBT) have been recommended as an adjunctive approach in the treatment of positive and persistent positive symptoms in TRS [28]. While this form of therapy is an interesting avenue to reduce positive symptoms in people suffering from TRS, results are mitigated, hence why new therapeutic strategies are currently emerging [29].

### 1.2. Current Innovation: Virtual Reality Therapy

Virtual reality therapy (VRT) for patients suffering from TRS is also known as avatar therapy (AT). This psychotherapeutic approach, developed by Julian Leff and his team in 2008, involves the use of an immersive virtual reality system in which TRS patients interact with an avatar, which virtually represents their main persistent auditory verbal hallucination while being controlled and animated by the therapist [30].

Numerous studies have shown that AT is useful in reducing auditory and verbal hallucinations [31,32]. AT is also a psychotherapeutic treatment developed at the Centre de Recherche de Institut universitaire en santé mentale de Montréal (CR-IUSMM). Studies are undergoing to evaluate its efficacy compared to that of CBT [32]. Nine weekly therapy sessions are planned as part of the therapeutic process, most of which are derived from other relational therapeutic approaches and CBT [32]. To accurately depict the patient’s own representation of their most upsetting speech hallucination (persistent auditory hallucinations; “voice”), the therapist and the patient work together to create an avatar during the first session using Unity software. The design of the avatar allows for the consideration of a wide range of traits, including gender, facial features, breadth, voice, and height. Patients then engage with the avatar during a portion of the following eight sessions while wearing a virtual reality headset. The therapist controls the animation of the avatar and operates an external speech modulator system to control their voice modulation.

Pilot studies examining the effects of AT showed that patients with TRS improved by significantly lowering the frequency of persistent auditory hallucinations as well as the distress associated with these symptoms, with large effect sizes [31,32]. Moreover, this therapy also significantly improved their quality of life [33]. To better document and analyze the content of the immersive portions (dialogues between the avatar and the patient), two main qualitative studies were conducted. Five key themes emerged from an initial qualitative investigation of the discourse elements of AT among the patients: emotional responses to voices, beliefs about voices and schizophrenia, self-perceptions, coping mechanisms, and aspirations [34]. Then, a second in-depth investigation by Beaudoin and her team provided further details. The verbatims (immersive session transcripts) of 18 patients who received AT were analyzed for content. Positive techniques (comprising six sub-themes) and confrontational techniques (comprising eight sub-themes) were the two main core interactions identified for the avatar [35]. The patients’ self-perceptions, emotional reactions, goals, coping strategies, and beliefs were all highlighted as five major themes comprising 14 sub-themes. Qualitative data are vast and insightful, and they can lead to the generation of novel hypotheses [35]. However, they lacks the quantitative equivalent required to identify the precise components of therapy that may help patients achieve favorable outcomes.

Considering the above limitations and potential human biases involved in the classification of verbal interactions, automated classification approaches have been attempted to analyze new verbatims of patients who underwent AT by classifying every interaction into one of the sub-themes identified by Beaudoin and her team. Several machine learning classifiers were compared [36]. In the end, the linear support vector machine classifier (LSVC) performed best for classification of therapeutic interactions taking place in AT [37].

AT being a novel approach for which access is currently limited to research participants, a better understanding of the therapeutic outcomes and predictors of such outcomes could be helpful when assessing which patients will benefit from the therapy. Considering that therapeutic components (i.e., verbal interactions between the patient and their avatar) can be qualitatively and quantitatively assessed, it could be beneficial to assess their predictive power of the reduction of persistent auditory hallucinations and consequently better personalize the treatment of TRS patients.

### 1.3. Precision Medicine Using Predictive Approaches

Modern medicine encompasses several precision medicine avenues, including the use of artificial intelligence to conduct an array of tasks: helping clinicians establish diagnosis, choosing treatment plans, outcome prediction, etc. [38,39,40]. Although precision medicine lags in psychiatry compared to other areas of medicine, it has been highlighted as an avenue to help patients and clinicians in achieving personalization of treatment plans [41]. A recent example is the implementation of a crisis predictor from the exploration of 581 656 medical records for patients suffering from various psychiatric disorders [42]. The model, developed by Garriga and his team, predicted crises with a sensitivity of 58% and a specificity of 85%, achieving an area under the receiver operating characteristic curve of 0.797 and an area under the precision-recall curve of 0.159 [42]. As for treatment responses, a relevant review identified eight studies about patients suffering from depression in which implementations of machine learning models have shown good treatment response prediction (up to 80% accuracy), frequently surpassing usual regression techniques [43]. Although this literature is scarce, several reviews on the application of machine learning to psychiatry and psychology support the idea that this avenue could provide more personalized care for patients [44]. Such use of machine learning in VRT could provide an insight as to which patients are more likely to benefit from this approach earlier or later in their recovery process. Moreover, such tools could also help therapists in planning their immersive sessions more effectively and ultimately conduct better tailored therapeutic sessions to help each patient in achieving favorable outcomes.

### 1.4. Objective and Hypothesis

By combining a classification model with a regression model, the aim of the study is to predict patients’ therapeutic outcome based on the interactions taking place in their first AT therapeutic session. The classification model is used to classify a verbatim into the right interaction themes, and the regression model is used to determine the predictive value of the newly annotated verbatim. Given the prior use of automated classification algorithms in virtual reality therapy, along with the binary nature of the therapeutic outcome, it is hypothesized that this process can be employed to predict a patient’s therapeutic outcome. To the best of our knowledge, this is the first attempt to predict the outcome of a patient in psychotherapy based on the content of their interactions with their therapist.

## 2. Materials and Methods

### 2.1. Participants and Recruitment

Data from participants involved in previous studies were used for the purpose of this investigation. These individuals were signed up for the clinical trial with the NCT03585127 identifier that was listed on ClinicalTrials.gov (accessed on 12 September 2023) [32]. All of them underwent six to ten one-hour psychotherapy sessions, eight of which involved interaction with an avatar that represented their auditory verbal hallucinations, while the creation of the avatar took place during the first session. The immersive portion of the therapeutic sessions between the patient and the avatar lasted between 15 and 50 min. Recruitment occurred at the CR-IUSMM between 2017 and 2022; participants were either referred by their treating team or self-referred. Inclusion criteria included an age of 18 or older and a diagnosis of TRS, characterized by a persistent auditory hallucination despite two or more trials of dopaminergic antagonists. The dataset consisted of treatment interventions for 18 patients, and the prediction power was tested using data from 17 other participants not included in the initial dataset. Therefore, for these 17 patients, the therapeutic outcome was known to the therapists but was unknown to the predictive algorithm.

### 2.2. Dataset: Corpus of Avatar Therapy and Features

**Figure 1:**
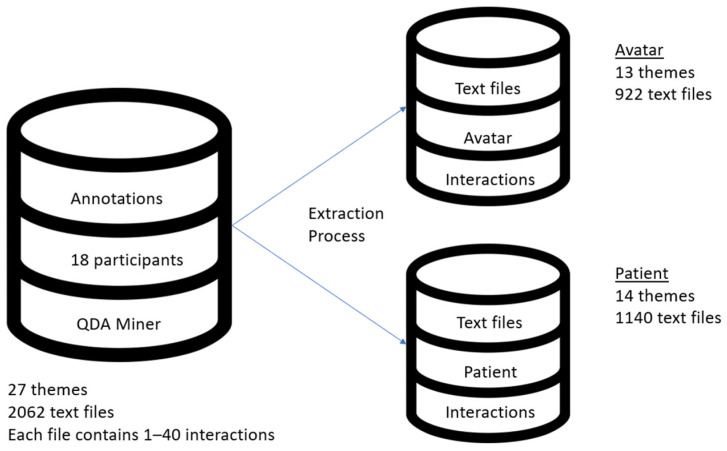
AT dataset and visual representation of Avatar and Patient conceptual databases.

A dataset comprising a total of 162 handwritten treatment transcripts of 18 patients who received VRT between 2017 and 2020 at our institution, corresponding to up to 10 therapy sessions per patient, was developed. The transcripts were written in Canadian French. The 27 themes listed in Beaudoin et al. 2021 were used to hand annotate transcripts [35]. This qualitative analysis of AT was carried out in previous research. Every one of the distinct interactions was individually coded by two study assistants. The same two research assistants cross-validated the robustness of the coding grid. Annotations were performed using the qualitative data analysis program QDA Miner version 5 (Provalis Research) [45]. Then, these were retrieved as text files from QDA Miner and categorized under two conceptual databases, Avatar and Patient, in order to optimize the automatic categorization. These text files contained between one and forty interactions of the same topic. The conceptual datasets were created in accordance with Figure 1’s representation.

In this study, the individual interactions, presented in Table 1 for the avatar and the patient, were used as features for the classification algorithm and the predictive algorithm. Both implementations are presented below.

### 2.3. Overview of the Predictive Approach

**Figure 2:**
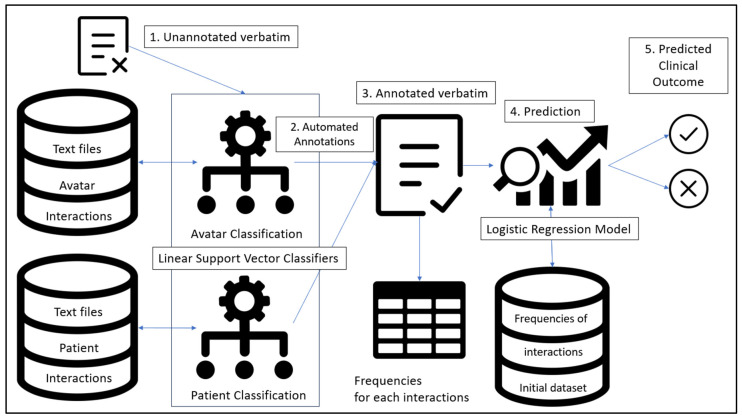
General flow and elements of the combination of LSVC for each conceptual database combined with a regression algorithm.

This study combines an automated classification algorithm with a logistic regression. The classification model was trained based on the AT dataset for each conceptual database. Then, an unannotated verbatim for the first immersive session of a participant who previously underwent AT (but unknown to the AT dataset) was presented to the classification model. Once automated classification of each interaction between the avatar and the patient was achieved, the frequency for each interaction was compiled and passed through the logistic regression model. A prediction of the outcome could then be achieved. The overall flow of this predictive approach is presented in Figure 2.

### 2.4. Automated Classification of Verbatims

#### 2.4.1. Previous Work

A support vector machine (SVM) was implemented as per previous work on avatar therapy [37]. This machine learning method is used for both regression and classification problems. It operates by identifying the ideal hyperplane in a high-dimensional feature space that best divides several classes [46]. This hyperplane is set up to maximize the distance (margin) between the classes, which enhances the model’s ability to generalize to new data. SVMs are a well-liked option for text classification jobs because of their capacity to handle high-dimensional, sparse, and non-linear data [46].

In this study, a linear form of SVM was combined to a term frequency-inverse document frequency statistic (TF-IDF). Compared to various SVMs with a tokenizer combination, a TF-IDF performs best with text categorization [47]. The TfidfVectorizer class, available in the Scitkit-Learn open library, was chosen for the TF-IDF tokenization as it allows for the conversion of raw text (extracted interactions from interview transcripts) into numerical vectors [48]. Stop-words can be accounted for by customizing vectorizers. The features were expected to be linearly separable since the classification categories were created so that text entities would be divided based on their inherent qualities, which are fundamentally distinct and specified in Beaudoin et al. (2021). A GridSearchCV (GSCV) was employed to guarantee the LSVC algorithm’s optimal performance and to improve search tactics [48]. The benefit of a GSCV is that it allows the user to test for various hyper-parameters and cross-validate the LSVC’s classification to find the optimal set of LSVC parameters and TfidVectorizer parameter variables. The full implementation details, including implementation parameters and hypertuning, can be found in Hudon et al. [37].

#### 2.4.2. Linear Support Vector Classifier

The LSVC employs a linear kernel as opposed to regular SVM [49]. A kernel is a mathematical function that transforms data into a higher-dimensional feature space and is used in several machine learning techniques [50]. Kernels are crucial to algorithms’ capacity to solve complex problems that might be difficult or even impossible to handle in the original input space [50]. When the data can be separated linearly, a linear kernel is thus applied. Scikit-Learn’s SVC class of the SVM library, which has the specification to use a linear kernel, is the implementation of the SVC used in this work [48].

### 2.5. Prediction of Patient’s Outcome

In this study, the outcome was measured based on the change in auditory hallucinations, measured using the Psychotic Symptom Rating Scales (PSYRATS) auditory hallucinations subscale [51]. The PSYRATS is a clinical evaluation instrument used to gauge how severe and specific psychotic symptoms are in psychotic individuals. The auditory hallucinations subscale is comprised of 11 items: frequency, duration, controllability, loudness, location, amount and degree of negative content, severity and intensity of distress, beliefs about the origin of voices, and disruption [51]. A participant who experienced a decrease of 20% in the PSYRATS auditory hallucination subscale was defined as being a good responder, whereas other participants were referred to as non-responders.

### 2.6. Data Analysis and Validation

#### 2.6.1. Training and Cross-Validation

For each conceptual database, a partitioning method was implemented, with 70% of the annotated documents being used to train the LSVC and the remaining 30% being used for testing. The goal was to determine a statistical likelihood for the LSVC, represented by a classification predictive score, which would indicate how well an interaction might be classified. To follow suggested design practices, the training and testing sets were purposefully kept apart [52]. The predictive score reflects the average accuracy as determined by the F1-Score. The K-Fold model from the Scikit-Learn module was used to build a tenfold cross-validation strategy for both the logistic regression algorithm and the linear support vector algorithm.

#### 2.6.2. Classification Analysis

Information on the classification performance of each topic, including accuracy, recall, and F1-Score for each method, was gathered using the Classification Report tool from the Scikit-Learn metrics module [48]. The F1-Score depicts the accuracy of theme categorization, recall of the sensitivity of the prediction, and precision of the positive predictive value. To provide a comprehensive evaluation of classification accuracy, the F1-Score, a widely used metric in text classification, finds a compromise between precision and recall [53]. Therefore, the harmonic mean of recall and accuracy is the F1-Score [53].

### 2.7. Prediction Analysis

Considering the binary outcome of virtual reality therapy (good responder versus non-responders), a logistic regression was implemented. The interactions between avatar and the patient as defined above were used as features to determine the outcome of the regression. The LogisticRegressionCV class, which is a logistic regression with build-in cross-validation from the Scikit-Learn library, was used [48]. The adjusted R2 score was representative of our predictive score. A score of 1 would indicate that the model explains all the variation of the dependent variable around its mean compared to a score of 0, which means that the model does not explain at all the observed variations. Collinearity between the different variables was accounted for in the logistic regression algorithm by providing the variance inflation factor (VIF) for each feature. 

To build the logistic regression model, the average frequency for each type of interaction for the first set of participants was used to construct a second dataset used for predictive purposes. This dataset contained all the frequencies of the interaction themes of the 18 participants who previously completed AT. Considering that the interaction themes are the features of the predictive model, this approach is needed to predict the potential therapeutic outcome of a newly annotated verbatim. The data used for this are available in Supplementary Material Table S1. 

Finally, to predict patients’ outcomes, automatically annotated verbatims of the first immersive session of 17 participants (second set of participants) were used. The frequency of each type of interaction was calculated and used by the model to conclude if the participant was forecasted to be a good responder or a non-responder. Statistical significance is defined by a likelihood ratio *p*-value smaller than 0.05 [54].

## 3. Results

### 3.1. Sample Characteristics

Interactions taking place in the verbatims of 18 patients were used to construct the initial interaction dataset, from which interaction frequencies were used to make the prediction of the outcome. The characteristics of the sampled patients can be found in Table 2.

### 3.2. Performance of the Classification Algorithm

The average performance of the LSVC for the automatic annotation of the verbatims of the second set of participants can be found in Table 3. The precision score ranges from 0.62 to 0.67, the recall ranges from 0.58 to 0.65, and the F1-Score ranges from 0.60 to 0.65. Classification scores for participants 007 and 016 are the lowest, whereas the average accuracy score for annotation is 63% (as per the F1-Score). The classification of avatar themes performed better than patient interaction themes (70% versus 62%). Sample performances for each theme and class balances for each feature are found in Supplementary Material Table S2.

### 3.3. Performance of the Predictive Algorithm

The logistic regression model achieved an adjusted R2 performance of 0.736 with a likelihood *p*-value of 0.04. From the first immersive session verbatim of the 17 participants, a total of 15 had a predicted outcome corresponding to their true outcome (88.2% accurate predictions). Errors occurred for participants 003 and 016. Amongst the previously identified 27 themes, 16 were selected for the model when accounting for collinearity and relevancy. Coefficients of the features as well as model performances are found in Appendix A. Logistic regression score and outcomes are presented for each participants in Table 4.

## 4. Discussion

This study aimed to combine a classification model with a regression model to predict patients’ treatment outcome based on interactions in their first AT immersive session. The verbatims (first immersive session) of 17 participants who previously underwent AT were automatically annotated based on a corpus from previous participants of AT, and a prediction was effectuated based on the frequency of each type of interaction that took place during the first VR session. As a result, the combination of the models predicted accurately the outcome of 15 out of 17 participants with an average accuracy score for the automated annotations of 63%.

Prediction of psychotherapeutic outcome is a potentially interesting avenue to personalize treatments for patients suffering from severe mental illnesses. However, prior works on the topic of predicting patient outcomes using patient risk factors and demographics were mostly inconclusive [55,56]. Patient factors alone cannot be the sole elements to predict patients’ outcomes as therapeutic processes are highly dependent on the therapeutic alliances and patient–therapist interactions. A five-year longitudinal study protocol highlighted the importance of considering the therapist’s interpersonal skills in relation with the outcome of the psychotherapy [57]. Other elements of the psychotherapeutic process are also in the process of being modeled. For example, a recent study trained several machine learning algorithms on patients’ self-reported side effects of psychotherapy to predict performances of the psychotherapeutic outcomes. They achieved an accuracy of 79.7% using Random Forest-based machine learning classifiers [58]. However, such implementation of prediction classifiers is to be used with caution as numerous biases might be implied such as class imbalances and the definition of the therapeutic outcome in their context of relevance. When compared to other fields of medicine, where terms (e.g., signs and symptoms) may be used to help categorization, psychotherapy such as AT often utilizes a larger range of words and contextual sentences. This might explain why the automated classification does not reach perfect accuracy. 

As for the predictive performance, few studies address predictive indicators of therapeutic outcomes based on therapeutic interactions. However, a recent literature review on the potential uses of machine learning to predict responses to CBT for different mental health disorders identified algorithm performances ranging from 67.3% up to 87.0% [59]. The performance of our model (88.2%) may differ from this range of performances because AT differs from CBT and uses a protocolized approach for patients suffering from TRS. However, the complexity of TRS and the variety of interactions between the therapists and the patients might account for the lower classification scores which is often observed when the elements of the corpus are overlapping or imbalanced. Another explanation could be that AT is based on an important role-playing relationship between the therapist and the patient, necessarily less linear in its approach, which could explain the low classification accuracy.

### Limitations

The performance trend for the LSVC is to be re-evaluated when additional patients are added to the dataset. Of note, transcripts used for this study’s analysis were written in Canadian French, and consequently, finding vectorizers that included stop-words particularly for the Canadian French language was challenging. Stop-words are terms that are often not included in the tokenization process because their meanings are either vague or unimportant. The accuracy of the analysis could have been impacted by the lack of suitable stop-words for Canadian French, which could lead to irrelevant terms being included in the word vectors and distorting the final findings. Finally, it is of importance to note that the performance of the regression algorithm was based on a limited dataset which might affect its performance and is dependent of the classification algorithm.

## 5. Conclusions

To conclude, this study demonstrated that classification algorithms such as LSVC can be combined with a predictive algorithm to predict patients’ outcomes for AT. Out of 17 participants unknown to the original dataset, the outcomes of 15 were accurately predicted based on the frequency of their interactions during their first immersive session. Automated classifications of the interactions taking place during the immersive session achieved performances comparable to previous studies on the subject. These results present an interesting avenue for the personalization of patients’ experiences with AT as the therapist might use this insight between the immersive sessions to help prepare their next sessions to enhance patients’ likelihood of achieving a favorable outcome. This could be carried out by identifying interactions linked to a positive outcome and encouraging such interactions. To the best of our knowledge, this is the first implementation of a predictor based on content elements of the therapeutic process. This opens the door to future studies to explore the possibility of using such classifiers in different psychotherapeutic contexts or by mixing potential predictive elements such as emotional content, therapeutic alliance, and patients’ characteristics.

## Figures and Tables

**Table 1:** Summary of interactions for the avatar and the patients in VRT as defined by Beaudoin et al.

Category	Theme	Definition	Verbatim Example (Translated from French to English)
Avatar themes			
Confrontational techniques	Threats	The avatar expresses an intention that is detrimental to the patient.	“I will haunt your family”
	Accusations	The avatar accuses the patient of having done or thought something. Can also include comments with an accusatory connotation.	“You are always complicating everything”
	Affirmations of omnipotence	The avatar affirms its existence or its omnipotence.	“I am the strongest”
	Incitements, orders	The avatar encourages or orders the patient to take actions or to think in a certain way.	“Go play with the machines, go spend all your money”
	Provocation	Any words of the avatar which can lead the patient to react, including but not limited to, insults, belittlement, and irony. Generally corresponds to what the patient reports to hear on a daily basis.	“I see that you have not gained any self-confidence”
	Manipulation using positive emotions	The avatar expresses a positive emotion or suggests one in a way that leaves no room for interpretation.	“I am having fun with you” (in response to “I do not want anything to do with you because you are mean”)
	Manipulation using negative emotions	The avatar expresses a negative emotion or suggests one in a way that leaves no room for interpretation.	“I am not happy you are telling me to go way”
Positive techniques	Reinforcement	The avatar encourages the patient to assert themselves or use coping mechanisms, or acknowledges that the patient is starting to do so.	“You did that all alone, too” (in response to “I listened to the tapes again, and I think I was courageous”)
	Reconciliation	The avatar suggests that the patient should make peace with them, or agrees to do so.	“Do we make peace?”
	Questions about self-perceptions	The avatar questions the patient about their perceptions of themselves (e.g., qualities)	“How do you explain that your father finds you courageous?”
	Questions about coping mechanisms	The avatar questions the patient about their coping mechanisms (self-defense, prevention, wishes), or makes comments leading the patient to question their coping mechanisms.	“How could you make me leave? How could you make sure I will not come back?”
	Questions about beliefs	The avatar questions the patient about their beliefs (origin of the voice, intentions of the voice, mental illness), or makes comments leading the patient to question their beliefs.	“I am simply repeating what you think of yourself”
	Empathetic listening, empathy	The avatar makes empathetic remarks, ask questions, agrees with what the patient says, or reformulates the patient’s words.	“Do you have an example?”
Patient themes			
Emotional response	Positive reactions	The patient expresses a positive emotion while talking with the avatar.	“I am happy”
	Negative reactions	The patient expresses a negative emotion while talking with the avatar.	“I cry almost every day because I am sick of this”
Beliefs	Maliciousness of the voice	The patient attributes malicious intents to the avatar.	“You are telling false stories”
	Omnipotence	The patient states that the voice is omnipotent, powerful, or beyond their control.	“I have always been sick because of you”
	Other beliefs	The patient expresses beliefs about the origin of the voice or about their mental illness.	“I do not think you can do that because you are not real”
Self-perceptions	Self-appraisal	The patient makes self-enhancing comments about themselves.	“You just told me I am a liar, but that is not true, I am honest”
	Self-deprecation	The patient makes demeaning comments about themselves.	“I have qualities, but there are not many qualities, they are rare…”
Coping mechanisms	Self-affirmation	The patient is assertive and demonstrates good self-confidence, usually in response to an affirmation from the avatar.	“I am not giving you a choice”
	Negation	The patient disagrees with an affirmation from the avatar.	“You will not have control over me”
	Counter-attack	The patient is countering an attack from the avatar.	“It’s getting hot, but it’s going to get even hotter if you stay in my head, you’re going to get hotter. It’s not me who’s going to make you burn, it’s the inner strength of my good will that’s going to make you burn.”
	Approbation	The patient agrees with an attack from the avatar.	“Well, well, alright” (in response to “things are going to go badly for you”)
Aspirations	Reconciliation with the voice	The patient expresses their wish to make peace with the avatar, for example by offering to make a deal.	“In the evening, you can come and talk to me. But during the day, leave me alone.”
	Disappearance of the voice	The patient expresses their wish that the voice or avatar disappears, go away, or leaves them alone.	“Get out of my head”
	Prevention strategies	The patient establishes cognitive, behavioral, or affective strategies to cope with the distress associated with the voice.	“I will feel OK and I will think of the good god of love that I love on earth”

**Table 2:** Characteristics of sampled patients for the first set of participants included in previously published studies. N = 18.

Characteristics	Value (N = 18)
Male sex (N)	15 (83%)
Age (mean ± SD in years)	40.8 ± 12.1
Education (mean ± SD in years)	13.2 ± 3.4
Caucasian ethnicity (N)	16 (89%)
Clozapine use (N)	11 (61%)

**Table 3:** Average performances of each participant on the Avatar conceptual database for the metrics. N = 17.

Participant	Precision	Recall	F1-Score
Participant 001	0.65	0.63	0.63
Participant 002	0.67	0.65	0.65
Participant 003	0.63	0.61	0.61
Participant 004	0.65	0.63	0.63
Participant 005	0.65	0.63	0.63
Participant 006	0.64	0.63	0.63
Participant 007	0.62	0.58	0.60
Participant 008	0.65	0.65	0.65
Participant 009	0.65	0.63	0.63
Participant 010	0.65	0.63	0.63
Participant 011	0.64	0.63	0.63
Participant 012	0.64	0.61	0.62
Participant 013	0.65	0.63	0.63
Participant 014	0.65	0.63	0.63
Participant 015	0.65	0.63	0.63
Participant 016	0.62	0.58	0.60
Participant 017	0.65	0.63	0.63
Average scores	0.65	0.63	0.63

**Table 4:** Comparisons of the true outcome to the predicted outcome for the verbatim of the first immersive session of the 17 participants.

Participant	True Outcome	Predicted Outcome	True Logistic Regression Score
Participant 001	Good responder	Good responder	1
Participant 002	Good responder	Good responder	1
Participant 003	Good responder	Good responder	1
Participant 004	Non-responder	Good responder	0
Participant 005	Non-responder	Non-responder	0
Participant 006	Non-responder	Non-responder	0
Participant 007	Non-responder	Non-responder	0
Participant 008	Good responder	Good responder	1
Participant 009	Non-responder	Non-responder	0
Participant 010	Good responder	Good responder	1
Participant 011	Good responder	Good responder	1
Participant 012	Non-responder	Non-responder	0
Participant 013	Good responder	Good responder	1
Participant 014	Good responder	Good responder	1
Participant 015	Non-responder	Non-responder	0
Participant 016	Non-responder	Good responder	0
Participant 017	Non-responder	Non-responder	0

## Data Availability

The datasets generated and/or analyzed during the current study containing patients’ verbatims are not publicly available due to patients’ privacy but are available from the corresponding author on reasonable request.

## Supplementary Materials

The following supporting information can be downloaded at: https://www.mdpi.com/article/10.3390/jpm13121660/s1, Table S1: Frequencies of all avatar and patient themes across avatar therapy; Table S2: Sample performances for each theme and class balances for each feature.

## Appendix A

**Table A1:** Logistic regression model’s performances.

Model	Logistic Regression
Pseudo R-Squared	0.736
Likelihood ratio *p*-value	0.04
intercept	−4.651781763166642
coefficients:	
Self-appraisal (patient)	0.04883651593641261
Self-affirmation (patient)	0.02567784838758098
Beliefs (avatar)	−0.09561273366103458
Negative (patient)	−0.11364269902464413
Psychotherapeutic interventions (neutral)	0.010654286427180373
Other beliefs (patient)	0.029167922584813755
Provocation (avatar)	0.1866938254993158
Negation (patient)	0.1231509841333093
Prevention (patient)	−0.31748282158545243
Accusations (avatar)	−0.12466383099943076
Positive (patient)	0.4499983951894208
Questions about coping mechanisms (avatar)	−0.35225305832560383
Counterattack (patient)	0.10198904940934386
Questions about self-perceptions (avatar)	0.4672076538702732
Reinforcement (avatar)	−0.005131226285317009
Maliciousness of the voice (patient)	−0.154875181322441
