# Supplementary material for: Enhancing Predictive Power: Integrating a Linear Support Vector Classifier with Logistic Regression for Patient Outcome Prognosis in Virtual Reality Therapy for Treatment-Resistant Schizophrenia
Source: J Pers Med. 2023 Nov 28;13(12):1660. doi: 10.3390/jpm13121660 (PMC10744538; doi:10.3390/jpm13121660)
Supplement: Supplementary file 1 [file jpm-13-01660-s001.zip › TableS2.docx]

**Table S2**. Sample performances for each theme and class balances for each feature

| **Avatar theme** | **Precision (VPP)** | **Recall (sensitivity)** | **F1-score (specificity)** | **Sample test size** |
| --- | --- | --- | --- | --- |
| Accusations | 0.67 | 0.53 | 0.59 | 30 |
| Omnipotence | 0.53 | 0.73 | 0.62 | 11 |
| Beliefs | 0.76 | 0.59 | 0.67 | 32 |
| Active listening, empathy | 0.76 | 0.8 | 0.78 | 20 |
| Incitements, orders | 0.67 | 0.91 | 0.77 | 11 |
| Coping mechanisms | 1 | 0.75 | 0.86 | 16 |
| Threats | 1 | 0.91 | 0.95 | 11 |
| Negative emotions | 0.72 | 0.87 | 0.79 | 15 |
| Self-perceptions | 0.65 | 0.65 | 0.65 | 23 |
| Positive emotions | 0.9 | 0.6 | 0.72 | 15 |
| Provocation | 0.43 | 0.71 | 0.54 | 14 |
| Reconciliation | 0.73 | 0.73 | 0.73 | 15 |
| Reinforcement | 0.7 | 0.78 | 0.74 | 18 |
| Average scores | 0.73 | 0.71 | 0.706 | 231 |

| **Patient themes** | **Precision (VPP)** | **Recall(sensitivity)** | **F1-score (Specificity)** | **Sample test size** |
| --- | --- | --- | --- | --- |
| Approbation | 0.15 | 0.14 | 0.15 | 14 |
| Self-deprecation | 0.32 | 0.75 | 0.44 | 8 |
| Self-appraisal | 0.65 | 0.6 | 0.63 | 25 |
| Other beliefs | 0.62 | 0.58 | 0.6 | 26 |
| Counterattack | 0.5 | 0.62 | 0.56 | 16 |
| Maliciousness of the voice | 0.5 | 0.42 | 0.45 | 12 |
| Negative | 0.6 | 0.58 | 0.59 | 31 |
| Negation | 0.95 | 0.56 | 0.7 | 34 |
| Omnipotence | 0.54 | 0.58 | 0.56 | 12 |
| Disappearance of the voice | 0.83 | 0.76 | 0.79 | 25 |
| Positive | 0.71 | 0.88 | 0.79 | 17 |
| Prevention | 0.75 | 0.75 | 0.75 | 32 |
| Reconciliation of the voice | 0.55 | 0.75 | 0.63 | 8 |
| Self-affirmation | 0.58 | 0.60 | 0.59 | 25 |
| Average scores | 0.65 | 0.65 | 0.62 | 285 |
